# P71 - Shared care and implementation of pediatric clinical pathway

**DOI:** 10.1186/2045-7022-4-S1-P126

**Published:** 2014-02-28

**Authors:** Mette Sørensen Langfrits

**Affiliations:** 1 Aarhus University Hospital, Aarhus, Denmark

## Background

Asthma is the most common chronic disease among Danish children and the aim of the treatment should be total control and no symptoms. Successful asthma management involves guideline-based treatment and regular follow-up. The international guidelines from "Global Initiative for Asthma" (GINA) are implemented as a clinical pathway and can improve intersectional collaboration on children with asthma, which is based on a shared responsibility of the treatment between general practitioners and pediatricians called Shared Care.

## Aim

1. The well-controlled asthmatic children have to be followed in general practice and asthmatic children without control have to be followed at the pediatrics department with defined intervals (based on evaluation of symptoms, lung function, treatment and compliance.)

2. The proportion of children with well-controlled asthma will increase.

3. We wish to show favorable changes in the use of asthma medication.

4. Children with asthma will get a higher quality of life.

## Methods

Follow-up study from 1^st^ of April 2011 to 31th of April 2014, with the inclusion of asthmatic children with a validated diagnosis aged 0-15 years followed at the out-patient clinic at the pediatrics department at a regional hospital and by 100 GPs in the area. The total number of patients in the area is estimated to 3000.

## Intervention

Implementation of a clinical pathway and treatment guide. Data is obtained from GP´s and the outpatient clinic, a regional prescription database and through questionnaires in the form of Paediatric Asthma Quality of Life Questionnaire (PAQLQ (S)), PACQLQ and The Childhood Asthma Control Test (C-ACT).

## Perspectives

The project will hopefully provide significant documentation, which can be use nationally for the recommendations of the future organization of childhood asthma diagnosis, treatment and control.

